# Patient-related outcome of unstable pelvic ring fractures stabilized with a minimal invasive screw-rod system

**DOI:** 10.1186/s12955-017-0821-7

**Published:** 2017-12-22

**Authors:** Maximilian Kerschbaum, Nadine Hausmann, Michael Worlicek, Christian Pfeifer, Michael Nerlich, Paul Schmitz

**Affiliations:** Clinic of Trauma Surgery, University of Regensburg, Franz-Josef-Strauss Allee 11, 93053 Regensburg, Germany

**Keywords:** Pelvic ring fractures, Transiliac internal fixator, Patient-related outcome, Sf-36, EQ-5D

## Abstract

**Background:**

Clinical and radiological outcomes of operatively treated unstable pelvic ring fractures are well documented, whereas little is known about the patient’s related outcome. The purpose of this study is to evaluate the patient-reported outcome after minimal invasive treatment of pelvic ring fractures using the SF-36 and EQ-5D medical outcome scores.

**Methods:**

Patients with unstable pelvic ring fractures treated in our trauma department with a minimal invasive screw-rod system between 01/2004 and 12/2014 were included. Next to patient data (sex, age), injury related details (fracture type, additional injuries, Injury Severity Score (ISS)) as well as operation details (method, time to operation, general complications, adverse events associated with the surgical procedure, revision surgery, fracture reduction) were assessed. The patient related outcome was evaluated using the SF-36 and the EQ-5D score.

**Results:**

A total of 105 patients (57 men; 48 women; mean age 56 ± 21 years) were identified as candidates for the study. 60 patients completed the SF-36 and EQ-5D score after a mean follow-up of five years (60.5 months (14-142 months)). Of these patients 77% were multiply injured with a mean ISS of 26 ± 19. Within the respondent group 22% showed type B and 78% type C pelvic ring fractures. In 82% the dorsal pelvic ring fracture was stabilized using a minimally invasive transiliac internal fixator, in 18% an iliolumbar fixation was performed respectively. The mean physical component score of the SF-36 was 37.9 ± 12.0, the mean mental component score was 49.8 ± 12.5. The mean EQ-5D VAS reached 70.5 ± 24.4.

**Conclusion:**

Patients being multiply injured and treated with minimal invasive treated dorsal pelvic ring fractures were suffering more especially concerning physical domains compared to the healthy reference population. Nevertheless, the overall patient-related outcome is comparable to pelvic ring fractures in general.

**Trial Registration Number:**

Clinical Trial Registry University of Regensburg Z-2017-0878-3. Registered 22. July 2017. Retrospectively registered.

## Background

**Fig. 1:**
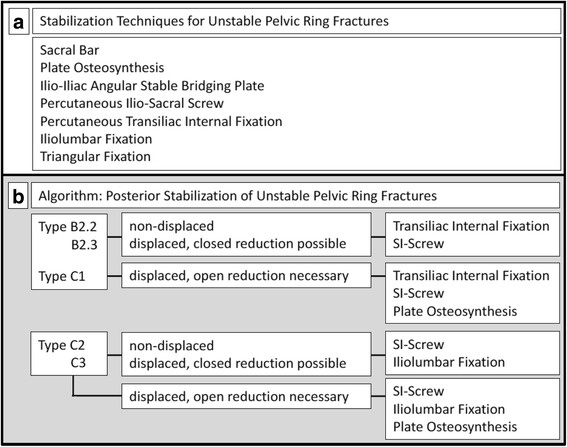
**a** Different surgical techniques and **b** Decision guidance (favored surgical techniques) to treat unstable type B and type C pelvic ring fractures

Unstable pelvic ring fractures are severe injuries often caused by high-energy trauma [1]. Patients suffering pelvic ring fractures frequently show associated injuries [1]. Stabilization with reconstruction of the pelvic ring anatomy is recommended in unstable and/or displaced pelvic ring fractures [2]. There are clinical and radiological results of various reduction and stabilization concepts already published (Fig. 1a) [3–9]. Especially minimal invasive techniques seem to be superior because of a high biomechanical and clinical stability with a minor risk of soft tissue injury, blood loss or neurovascular damage [8–10].

Especially the patient-reported outcome enables a better understanding of the global injury outcome and surgical procedures [2, 11, 12]. Therefore, over the recent years, health-related outcome measurement tools and life quality measurement instruments became more important for assessment of functional outcomes from different therapeutic interventions. The purpose of this study is to evaluate the patient-related outcome and life quality after minimal invasive treated unstable pelvic ring injuries using a screw-rod system. The SF-36 and EQ-5D outcome measurement tools were used to assess the health-related quality of life.

## Methods

**Fig. 2:**
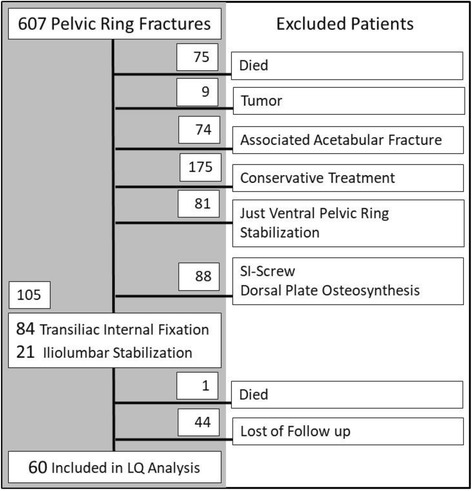
Flow chart: Inclusion and exclusion criteria for identified patients with unstable pelvic ring fractures

**Fig. 3:**
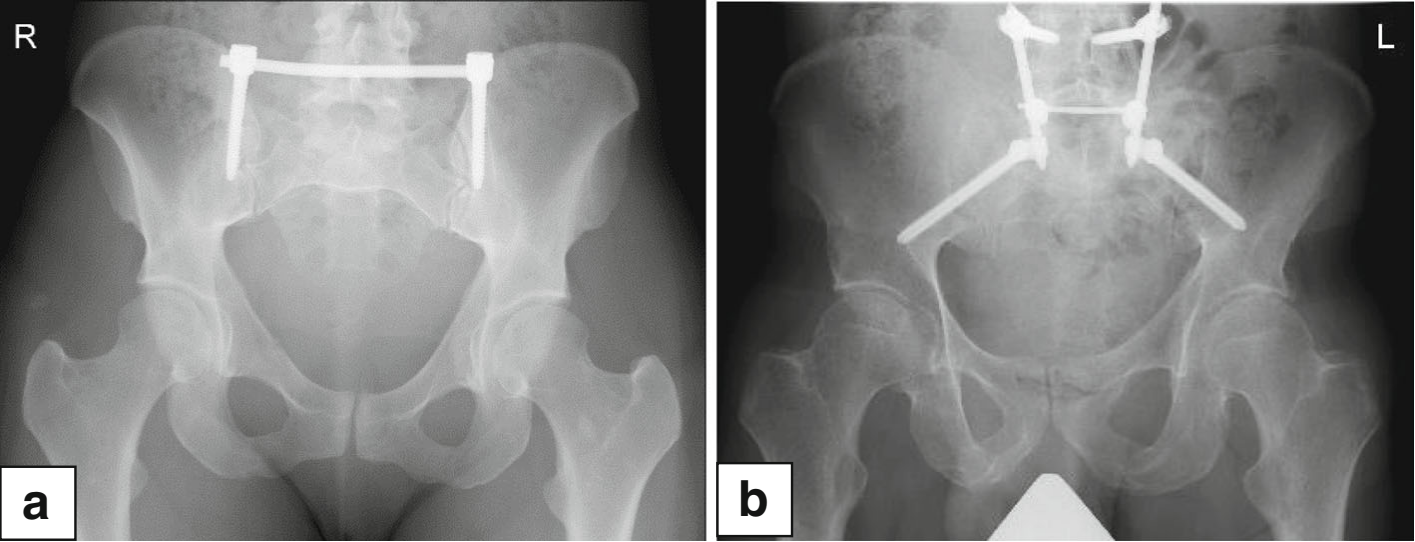
**a** Transiliac Internal Fixator; **b** Iliolumbar stabilization

Patients with unstable pelvic ring fractures treated with a minimal invasive screw-rod system in our trauma department between 01/2004 and 12/2014 were included in this study. Patients younger than 18 years were excluded. Detailed in- and exclusion criteria are shown in Fig. 2. Pelvic stabilization was performed using a minimal invasive transiliac internal fixator [9, 13] (Fig. 3a). In case of bilateral vertical unstable fracture, minimal invasive iliolumbar fixation was performed (Fig. 3b). Next to the patient related data (sex, age), injury related details (fracture type, additional injuries, Injury Severity Score (ISS)) as well as operation details (method, time to operation, complications, revision surgery, fracture reduction) were assessed. All fractures were classified based on plain X-rays and computer tomographic (CT) scans according the AO/OTA classification system [14].

Fracture reduction of the dorsal pelvic ring was measured on postoperative CT scans. The maximal displacement was measured and graded referring to the classification system published by Matta et al. [6].

The local institutional review board and ethic committee of the clinic approved the study. Only patients who agreed to participate the study by giving their written content were included.

### Quality of life (QoL)-instruments

The patient-related outcome and quality of life were assessed using the German SF-36 and EQ-5D medical outcome scores. Patients were contacted by telephone to ensure that they agree to participate the study. If patients were not reachable by phone, forms were sent to the last known address. The scores were posted to all patients with a minimum of 1 year follow-up.

### Sf-36

The German Short-Form 36 is an established instrument measuring health related life quality [15]. It consists of 36 questions and evaluates eight functional domains: physical function (PF), role physical (RP), bodily pain (BP), general health (GH), vitality (VT), social function (SF), role emotional (RE) and mental health (MH). Raw data transformation and summary score calculation were performed as described by Bullinger et al. [15]. Normative data from Germany (7525 persons) were used as reference [16].

### EQ-5D

The EuroQol-5D medical outcome score is a widely used generic life quality questionnaire, designed by the EuroQoL group [17]. It consists of five questions concerning the following functional domains: mobility, self-care, everyday life activities, pain/discomfort and anxiety/depression. Evaluation was performed using a preference-based method (time trade-off (TTO) as well as using the VAS-EQ-5D method [18]. The results were compared with normative data from Germany (2022 persons) [19].

### Statistics

Statistical analysis was carried out using SPSS software (SPSS Inc., Chicago, Illinois).

The chi-square independence test was performed to compare categorical variables, the independent t-test was used to compare continuous variables after determining the distribution was appropriate for parametric testing. *P*-values <0.05 were considered significant.

## Results

A total number of 105 patients (57 men; 48 women; mean age 56 ± 21 years) were identified. 60 patients (33 men; 27 women; mean age 51.6 ± 19.5) agreed participating the study and completed the SF-36 and EQ-5D score after a mean follow-up of five years (60.5 months (14-142 months)). All patients, who could have been contacted, were eligible to complete the QoL questionary. One patient died during inpatient stay of a septic multiorgan failure. 44 patients could not be contacted or traced. Overall, the response rate was 57.1%. Except for the mean age, the profile of the respondent and non-respondent group showed no significant differences. Patients’ characteristics of the respondent and non-respondent group are shown in Table 1.

**Table 1:** Patients characteristics of the respondent and non-respondent group

		Total (*n* = 105)	Respondent group (*n* = 60)	Non-respondent group (*n* = 45)	Significant differences
Age	[years] (min.-max.)	55.9 ± 21 (21–93)	51.6 ± 19.5 (21–89)	61.5 ± 21.8 (22–93)	0,016
Sex	[%] [n]	♂: 54.3 57	♂: 55.0 33	♂: 53.3 24	NS
Multiple injured	[%] [n]	73.3 77	76.6 46	68.9 31	NS
ISS^a^	Mean ±SD (min.-max.)	25 ± 19 (16–75)	26 ± 19 (16–75)	23 ± 20 (16–75)	NS
Head injury	[%] [n]	35.2 37	38.3 23	31.1 14	NS
Chest injury	[%] [n]	42.9 45	45.0 27	40.0 18	NS
Spine injury	[%] [n]	37.1 39	40 24	33.3 15	NS
Abdominal injury	[%] [n]	35.2 37	40.0 24	28.9 13	NS
Lower limb injury	[%] [n]	36.2 38	43.3 26	26.7 12	NS
Upper limb injury	[%] [n]	30.5 32	28.3 17	33.3 15	NS

^a^Injury Severity Score (ISS)

No significant differences between the respondent and non-respondent group concerning the fracture type were found (*p* > 0.05). The fracture types according to the AO/OTA classifications system [14] are shown in Table 2.

**Table 2:** Pelvic fracture classification of respondent patients according to the AO/OTA classification-system [14]

Fracture Type	Description		Cases n = 60
Type B	*Rotationally unstable, vertically stable*	[%] [n]	21.7 13
Type B1	Unilateral *Open book*	[%] [n]	1.7 1
Type B2	Unilateral *compression*	[%] [n]	15 9
Type B3	Bilateral	[%] [n]	5 3
Type C	*Rotationally and vertically unstable*	[%] [n]	78.3 47
Type C1	Unilateral	[%] [n]	55 33
Type C2	Bilateral *ipsilateral rotationally and vertically, contralateral rotationally unstable*	[%] [n]	10 6
Type C3	Bilateral	[%] [n]	13.3 8

In most of the patients concomitant injuries were found (Table 1). The mean ISS was 26 ± 19. There was a mean delay from the moment of injury to operative pelvic fixation of 4.5 days (range: 0-21d). 81.7% of the patients were treated with a minimal invasive transiliac internal fixator, while 18.3% of the patients received an iliolumbar fixation. A ventral osteosynthesis of the pelvic ring was performed in 88.3%.

### Complications associated to trauma and severity of injury

The majority of complications and adverse events were related to the trauma and the severity of injury (Table 3). While neurological complications occurred in 1.9% due to the injury, no neurological deficiency was caused by the operative treatment. Medical complications such as pneumonia, thrombosis, pulmonary embolism, acute respiratory distress syndrome (ARDS) occurred in 33.3% (Table 3). The mean ISS of patients who suffered a complication was 33 ± 20, whereas patients without any complication had a mean ISS of 19 ± 15 (*p* = 0.03). Medical complications occurred in patients with a high ISS score. The mean ISS in patients with medical complications was 40 ± 17 whereas the mean ISS in patients without medical complications was 19 ± 16) (*p* = 0.00). There was no significant difference detected concerning the complication rate of fracture type (*p* > 0.05).

**Table 3:** Adverse events associated to trauma and severity of injury

		Total (n = 105)	Respondent group (n = 60)	Non-respondent group (n = 45)	Significant differences
Neurological deficiency	[%] [n]	1.9% 2	3.3% 2	0%	NS
Bleeding	[%] [n]	5.7% 6	3.3% 2	8.9% 4	NS
Pneumonia	[%] [n]	6.7% 7	8.3% 5	4.4% 2	NS
Thrombosis/Pulmonary Embolism	[%] [n]	9.5% 10	3.3% 2	17.8% 8	0.013
ARDS	[%] [n]	4.8% 5	8.3% 5	0% 0	0.047
Others	[%] [n]	12.4% 13	13.3% 8	11.1% 5	NS

### Adverse events associated to the surgery

Surgical site complications were found in 11.7%. Revision surgery was necessary in 2.9% (Table 4).

**Table 4:** Surgical side complications and revision surgery

		Adverse events	Revision needed
Seroma	[%] [n]	1.9 2	
Hematoma	[%] [n]	1.9 2	
Pin tract infection	[%] [n]	2.9 3	
Deep wound infection	[%] [n]	1.9 2	1.9 2
Surgical induced hemodynamic bleeding	[%] [n]	0	
Surgical induced neurological deficiency	[%] [n]	0	
Pedicle screw malposition	[%] [n]	1 1	1 1
Ilium screw malposition	[%] [n]	1.9 2	
Secondary dislocation of the osteosynthesis	[%] [n]	0	

### Fracture reduction

In 78 patients the postoperative maximal displacement of the dorsal pelvic ring fracture was less than 2 mm. In twelve patients 2–4 mm were measured. Thirteen patients showed a maximal displacement of 5-10 mm, while two patients had a displacement greater than 10 mm.

### Quality of life (QoL):

The SF-36 health outcome score of the respondent group compared with normative data of German population is shown in Table 5. While patients after pelvic ring fractures show similar values concerning the mental health compared to the German reference group, physical domains after pelvic ring fractures are mostly affected.

**Table 5:** SF-36 medical outcome scores for pelvic fracture compared with a normative reference of a German population [16]

	Respondent group	German population (2013) [16]
Physical component score (PCS)	37.9 ± 12.0	51.4 (51.1–51.7)
Physical functioning	58,0 ± 32.8	86.6 (86.0–87.2)
Role physical	40.4 ± 43.2	82.1 (81.3–82.8)
Bodily pain	61.4 ± 27.6	74.8 (74.1–75.6)
General health	55.2 ± 22.4	69.3 (68.7–69.9)
Mental component score (MCS)	49.8 ± 12.5	49.3 ± 49.0–49.6
Vitality	49.9 ± 22.3	61.6 (61.0–62.1)
Social functioning	74.1 ± 29.7	86.1 (85.4–86.7)
Role emotional	72.8 ± 40.9	86.0 (85.3–86.6)
Mental health	69.2 ± 21.6	72.9 (72.4–73.4)

Results of the EQ-5D compared to normative data of a German reference group [19] are illustrated in Table 6.

**Table 6:** EQ-5D medical outcome score for pelvic fracture and normative data from a German population [18]

	Respondent group	German population (2013) [18]
EQ-5D preference based	77.3 ± 22.8	84.9 ± 16.5
EQ-5D VAS	70.5 ± 24.4	77.1 ± 17.8

**Fig. 4:**
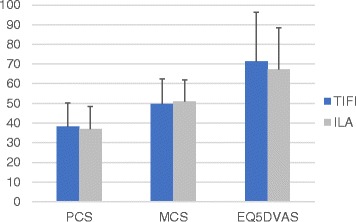
Difference of the physical component score (PCS), mental component score (MCS) and EQ-5DVAS score between patients with a minimal invasive transiliac internal fixator (TIFI) and patients with iliolumbar fixation (ILA)

**Fig. 5:**
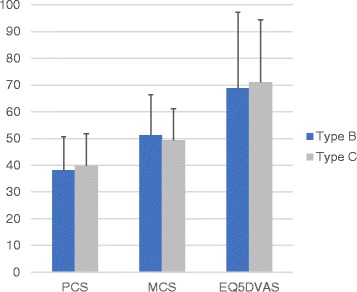
Differences of the Physical component score (PCS), mental component score (MCS) and EQ-5DVAS score between Type B and Type C fractures

Neither the SF-36, nor the EQ-5D health outcome score showed a significant difference(*p* > 0.05) between Type B and Type C fractures (Fig. 4) nor for the kind of treatment (TIFI vs. ILA) (Fig. 5). Furthermore, the QoL scores (SF 36, EQ-5D) could not detect any significant differences for the accuracy of fracture reduction (*p* > 0.05).

## Discussion

This is the first study investigating the patient-related outcome and life quality after minimal invasive treatment of the dorsal pelvic ring using a screw–rod system. Compared to previous published investigations on pelvic ring fractures, the large patient cohort (60 respondent patients) and the long-time follow-up (five years) are outstanding characteristics of our study**.**

In our trauma department (Level 1 trauma center), we established a treatment algorithm for unstable pelvic ring fractures as shown in Fig. 1b. Even so, the SI-screw fixation is the most common minimal invasive way to stabilize transforaminal- and transalar sacral fractures we favor the use of an internal fixation by a screw rod system since there is hardly any risk for ilium screw malposition. Furthermore, the time consuming necessity of an image intensifier to find the right corridor for SI-screws is much bigger than for ilium screws**.** Previous biomechanical and clinical trials confirmed a high stability and a low complication rate using a screw-rod system (transiliac internal fixator) to stabilize dorsal pelvic ring fractures [8–10]. Nevertheless, the transiliac internal fixator is not a common procedure to treat posterior pelvic ring instabilities and it has to be mentioned critically that a stabilization of an AO/OTA Type B fracture with an internal screw rod system is probably over utilized. It might get more relevant in treatment of fragility fractures of the pelvis since it is angular stable and can be cement-augmented.

Oliver et al. pointed out, that outcome data, especially concerning the QoL are indispensable for the planning of surgical strategies and clinical decision making [11].

The overall finding in this study is the lower health-related life quality compared to a reference population, especially in physical domains. Previous published investigations concerning the QoL after operatively treated pelvic ring fractures show similar results. Oliver et al. evaluated the quality of life of 35 patients after operatively treating pelvic ring fractures using the SF-36 medical outcome score. They also present a lower of health-related quality of life compared to the reference population [11]. Van den Bosch et al. evaluated 31 patients after operatively treated pelvic ring fractures with a mean follow-up of 2.9 years (35.6 months) and found lower values for the QoL compared to a Dutch reference group [20]. Borg et al. also investigated a substantially lower patients’ quality of life after surgical treatment of pelvic ring fractures compared to the reference population, despite good clinical and radiological outcomes [2].

Except the mean age, the profile of the non-respondent group showed no significant differences compared to the respondent group. All patients, who were contacted, were eligible to complete the QoL questionary. Nevertheless, the low response rate of the patients (57.1%) is a limitation of this study.

Another limitation of the study is, that it is not possible to distinguish to what extend a lowered QoL is attributable to the pelvic ring fracture in multiple injured patients. 75% of the investigated patients in our study were multiply injured with a mean injury severity index (ISS) of 26. While the mean ISS of the patient population of Oliver et al. was considerably lower than in this study (17) [11], Van den Bosch et al. evaluated patients with a mean ISS of 30 [20]. A direct comparison of the results to previous reported QoL results is only possible in a limited way, due to the above mentioned differences in injury patterns and severity.

Even so this study is the first study that investigates the dorsal pelvic ring stabilization via a minimal invasive surgical approach. A direct comparison of the QoL of this study’s patients to previous published data with other surgical approaches is due to the above mentioned points impossible.

As pointed out by Hernefalk et al. it is not possible for severe injured patients to retrospectively assess their preinjury QoL without over- or underestimating. This retrospective evaluation is not included in this study [21].

## Conclusion

In conclusion, minimal–invasive treatment of unstable pelvic ring fractures using a screw-rod system in multiple injured patients show comparable patient-reported outcome to previous published QoL data. Fracture type (Type B versus. Type C pelvic ring fracture) and the amount of stabilization (transiliac internal fixator vs. iliolumbar fixation) make no difference. Nevertheless, the patients reported outcome is substantially lower especially concerning physical domains compared to a healthy reference population.

## Abbreviations

ARDS: Acute respiratory distress syndrome
ASA: American Society of Anesthesiologists
BP: Bodily pain
CT: Computer tomographic
GH: General health
ILA: Iliolumbar Fixator
ISS: Injury Severity Score
MH: Mental health
PF: Physical function
QoL: Quality of live
RE: Role emotional
RP: Role physical
SF: Social function
TIFI: Transiliac Internal Fixator
TTO: Time trade-off
VAS: Visual Analog Scale
VT: Vitality
